# A Novel Pax5-Binding Regulatory Element in the Igκ Locus

**DOI:** 10.3389/fimmu.2014.00240

**Published:** 2014-05-23

**Authors:** Rena Levin-Klein, Andrei Kirillov, Chaggai Rosenbluh, Howard Cedar, Yehudit Bergman

**Affiliations:** ^1^Department of Developmental Biology and Cancer Research, Institute for Medical Research Israel-Canada, Hebrew University Medical School, Jerusalem, Israel

**Keywords:** V(D)J rearrangement, DNA methylation, B cell development, Pax5, somatic hypermutation

## Abstract

The Igκ locus undergoes a variety of different molecular processes during B cell development, including V(D)J rearrangement and somatic hypermutations (SHM), which are influenced by *cis* regulatory regions (RRs) within the locus. The Igκ locus includes three characterized RRs termed the intronic (iEκ), 3′Eκ, and Ed enhancers. We had previously noted that a region of DNA upstream of the iEκ and matrix attachment region (MAR) was necessary for demethylation of the locus in cell culture. In this study, we further characterized this region, which we have termed Dm, for demethylation element. Pre-rearranged Igκ transgenes containing a deletion of the entire Dm region, or of a Pax5-binding site within the region, fail to undergo efficient CpG demethylation in mature B cells *in vivo*. Furthermore, we generated mice with a deletion of the full Dm region at the endogenous Igκ locus. The most prominent phenotype of these mice is reduced SHM in germinal center B cells in Peyer’s patches. In conclusion, we propose the Dm element as a novel Pax5-binding *cis* regulatory element, which works in concert with the known enhancers, and plays a role in Igκ demethylation and SHM.

## Introduction

The B cell receptors (BCRs) are encoded in the mouse genome by the three immunoglobulin (Ig) loci, the IgH heavy chain locus, and the two light chain loci, Igκ and Igλ. In their germline conformations, the Ig loci do not give rise to functional proteins. It is only through a tightly regulated process of genome editing, termed V(D)J recombination, that the loci are reconfigured to allow transcription of an Ig gene in B cells. During the recombination process, the variable (V), diversity (D), and joining (J) segments are cleaved by the RAG complex and joined together into one continuous segment by the DNA repair machinery (1). Each rearrangement utilizes a single V, D (in the heavy chain), and J segment, and each B cell contains one productively rearranged heavy chain and light chain. In this way, B cells give rise to the multitude of antigen recognition specificities which constitutes the adaptive immune system.

The recombination of the different loci takes place in a developmentally staggered manner, with the IgH locus undergoing VDJ recombination first in the pro-B cell stage (2). Light chain rearrangement normally takes place only after a successful IgH rearrangement, which allows the cells to differentiate to the pre-B cell stage (3). In mice, the Igκ locus is the primary source for the BCR light chain and will undergo preferential rearrangement. The recombination of the different loci is kept tightly separated, despite the fact that the enzymatic machinery responsible for the processes is essentially the same and is present at both the pro- and pre-B cell stages. The light chain loci are maintained in an inaccessible chromatin state *via* epigenetic mechanisms prior to the pre-B cell stage, at which point they become available to the rearrangement machinery (3, 4). One such epigenetic mark is DNA methylation, a mark that is established at the Igκ locus during early embryonic development and which is hereditarily maintained during cell division (5). DNA methylation has been shown to block the activity of the rearrangement machinery *in vitro* (6). The Igκ locus undergoes selective demethylation at the pre-B cell stage, immediately prior to rearrangement (5, 7, 8). The rearranged Igκ allele is unmethylated from that stage onward, while alleles which do not undergo rearrangement remain methylated, even at the mature B cell stage. The low level of methylation is significant for an additional stage of Igκ editing during B cell development, namely for efficient somatic hypermutation (SHM), which will allow affinity maturation of the BCR in activated mature B cells (9). Methylated pre-rearranged Igκ sequences do not undergo proper SHM at this stage, whereas identical unmethylated sequences do (10).

The stage-specific transcription, rearrangement, and chromatin structure of the Igκ gene is mediated by regulatory sequences within and in proximity to the locus. The locus contains three characterized enhancers, including an intronic enhancer (iEκ) (11), located in the intron between the Jκ segments and the Cκ exon and two enhancers situated a few 1000 bases downstream of the Cκ exon, termed 3′Eκ (12) and Ed (13). These enhancers work in cooperation to promote stage-specific chromatin accessibility, DNA demethylation, V to J rearrangement, heightened transcription of the locus, and SHM in activated B cells, with different enhancers contributing to a varying extent to each one of these processes. iEκ and 3′Eκ have been implicated in promoting accessibility and rearrangement of the locus in pre-B cells (14–16), while 3′Eκ and Ed strongly effect the level of transcription and SHM in mature B cells (17, 18), neither of which is significantly affected by the deletion of iEκ (14, 18). All of the three enhancers contribute together to the demethylation of the locus (16, 19). Replacement of iEκ with the IgH intronic μ enhancer is enough to change the rearrangement timing of the locus to the earlier pro-B cell stage, showing that it is indeed these sequences which direct the temporal precision of the developmental program (20).

Other than the enhancers, there are a number of additional regulatory elements surrounding the Igκ locus, increasing the complexity of the regulation. The recently discovered HS10 element, which lies downstream of Ed, appears to mostly function in plasma cells. While itself being a weak enhancer, HS10 acts as a co-enhancer to strengthen the activities of 3′Eκ and Ed (21). A matrix attachment region (MAR) lies immediately adjacent to iEκ and mediates connections between the locus and the nuclear matrix (22).

The activities of the *cis* regulatory elements are mediated by various transcription factors, which either activate or repress the enhancer activity. Many of these transcription factors are master regulators of the B cell lineage, which are important for maintaining B cell identity, such as E2A and PU.1 that bind sites in iEκ and 3′Eκ and substantially contribute to the enhancer activity (23–27). However, binding of Pax5, a master regulator of B cell identity, has been surprisingly missing from these enhancers in mature B cells. While binding sites have been identified in 3′Eκ (24, 25, 28), as well as in K-I and K-II (29, 30), which are regulatory regions (RR) upstream of the Jκ segments, Pax5 plays an inhibitory role in this context and is released during the pre-B cell stage when the locus is activated. This is despite the fact that Pax5 itself is necessary for the active induction of the locus (31).

In this work, we characterize a region adjacent to the MAR/iEκ elements. We had previously identified this element as a participant in the demethylation process of the Igκ locus in cell culture and thereby designated it Dm (32). Here, we find that this element binds Pax5 in B cell stages from the pre-B cell stage and onward. It is necessary for demethylation of a pre-rearranged Igκ transgene, but deletion of the element in the endogenous locus does not affect the demethylation process. We find that the element contributes to efficient SHM of the Igκ locus, indicating that the Dm element functions at more than one stage of B cell development.

## Materials and Methods

### Mice

Targeted mice were backcrossed for 10 generations on a BALB/c background. Igκ^ΔDm/ΔDm^ mice were bred with wild-type (WT) BALB/c to produce Igκ^WT/ΔDm^ mice. Human Cκ knock-in mice (33) (gift from M. Nussenzweig) were bred with either WT BALB/c or ΔDm BALB/c to produce Igκ^WT/WT^Cκ^h/m^ and Igκ^WT/ΔDm^Cκ^h/m^ mice, respectively. Igκ^WT/ΔDm^ mice were bred with CAST/EiJ (Cast) mice (Jackson Laboratory) to produce BALB/c/Cast Igκ^WT/WT^ and BALB/c/Cast Igκ^ΔDm/WT/^littermates. Rag1^-/-^ mice (Jackson Laboratories) were bred onto a Cast background. Igκ^ΔDm/ΔDm^ were bred onto a C57BL/6 Rag1^-/-^ (B6) background containing the 3H9 IgH chain transgene (IgH^+^). CAST/EiJ Rag1^-/-^ mice were bred with C57BL/6 Rag1^-/-^ IgH^+^ either with or without a deletion of the Dm element, giving rise to B6/Cast Rag1^-/-^ IgH^+^ Igκ^ΔDm/WT^ and B6/Cast Rag1^-/-^ IgH^+^ Igκ^WT/WT^ mice, respectively. Mice were housed in specific pathogen-free conditions at the Hebrew University Medical School animal facility. Transgenic mouse lines Lκ, LκΔDm, and LκΔ70 were produced, using the constructs described in the Section “Targeting Constructs,” at the Hadassah Hospital Medical School Transgenic Unit. Two independent founder lines were produced for the Lκ transgene, four for the LκΔDm and three for the LκΔ70. The copy number of the transgene for each founder line varied from low (two insertions) to high (20 insertions) with most lines having a moderate number of insertions (four to eight insertions). All animal procedures were approved by the Animal Care and Use Committee of the Hebrew University of Jerusalem.

### Targeting constructs

The LκΔDm construct was prepared using the following steps; the 4.3-kb *Kpn*I–*Kpn*I fragment, containing VκJ5–Cκ sequence, was excised from the Lκ plasmid (34) and cloned into the *Kpn*I site of the Bluescript vector which was modified to destroy the polylinker *Xba*I site. The resulting pBSKpn2 plasmid was cut at unique compatible *Xba*I and *Nsi*I sites, and recirculized, resulting in the deletion of 930 bp *Xba*I–*Nsi*I Dm fragment from the JκCκ intron. The *Kpn*I–*Kpn*I Dm-deleted fragment was excised and reinserted into the Lκ plasmid, resulting in the LκΔDm construct.

The LκΔ70 construct was prepared using the following steps; the *Hin*dIII-blunt *Taq*I 2.6-kb fragment, containing the germline Jκ region, was cloned to *Hin*dIII–*Eco*RV sites of the Bluescript vector. Next, a blunted *Bst*EII–*Bgl*II 2-kb fragment containing the Cκ exon was cloned into blunted *Eco*RI–*Bam*HI sites of the previously described Jκ containing Bluescript vector to yield the p-Δ70 construct, which had the 70 bp *Taq*I–*Bst*EII deletion introduced into the *Hin*dIII/*Bgl*II 5.6-kb JκCκ germline sequence. The 1-kb intact intronic *Xba*I–*Hin*dIII region of pBSKpn2 plasmid (previously described, containing the *Kpn*I–*Kpn*I fragment from the Lκ plasmid) was replaced with *Xba*I–*Hin*dIII fragment bearing the 70 bp deletion, excised from p-Δ70. The 4.2-kb *Kpn*I–*Kpn*I fragment with the 70 bp deletion was excised from the resulting pBSKpn2 Δ70 plasmid and cloned back into the Lκ plasmid, replacing the original 4.3-kb *Kpn*I–*Kpn*I sequence, and yielding the LκΔ70 construct.

The ΔDm targeting vector was prepared as using the following steps; a short arm of homology (neo-SAH) plasmid was constructed by using *Ban*II (ends filled with Klenow) and *Nsi*I to excise the 1.25-kb MAR and Eiκ containing fragment from the pBκMAR plasmid. This fragment was cloned into the sticky *Xba*I and blunted *Pst*I sites of the Bluescript vector. This construct was next cut at the polylinker sites *Cla*I and *Eco*RI and used for insertion of the 1.26-kb *Not*I–*Xba*I loxP flanked neoR gene fragment from the pMMneoflox-8 plasmid (all restriction ends were made blunt by reaction with the Klenow fragment), a long arm of homology (TK-LAH) plasmid was constructed by excision of the 7.1-kb *Pst*I–*Pst*I germline Jκ–Cκ region containing fragment from pSPIg8 plasmid (ends were blunted by reaction with T4 polymerase) and ligation into the *Hin*dIII site (blunted with reaction with Klenow fragment) of pIC19R/MC1-TK. The final ΔDm targeting vector was produced by cloning of the 8.9-kb *Xho*I–*Sal*I fragment from TK-LAH into the neo-SAH *Sal*I polylinker site. Targeting strategy is illustrated in Figure S1 in Supplementary Material.

### Cells and cultures

All cells in this manuscript were grown in RPMI 1640 medium (Gibco) supplemented with 10% fetal calf serum, 2 mM l-Glutamine, 100 μg/ml penicillin, 100 μg/ml streptomycin, and 50 μM 2-mercaptoethanol. BaF3 cell medium was additionally supplemented with IL3 secreted by WEHI-3b cells. IL-7-dependent pre-B cell cultures used for chromatin immunoprecipitation (ChIP) analysis were performed as has been previously described (35). COP8 cells were transiently transfected with a Pax5 expression plasmid (gift from M. Busslinger) using the DEAE dextran method (36).

### Isolation and analysis of lymphoid cells from bone marrow and spleen

Bone marrow cells from femur and tibia bones were flushed out with PBS using a 25 G syringe needle. Spleens were disrupted and pulp dispersed in PBS. Erythrocytes were lysed with RBC lysis solution (Biological industries) and cells were washed. When indicated, cells were isolated on magnetic MACS columns (Miltenyi Biotech) by positive selection with either αCD19 magnetic beads or streptavidin magnetic beads and biotinylated αB220 (Miltenyi Biotech), according to the manufacturer’s instructions. Cell purity following isolation was assayed as <95% by flow cytometry (LSR II, BD Bioscience).

Cells from erythrocyte disrupted spleens and bone marrows were stained with the antibodies indicated and cellular composition was analyzed by flow cytometry (LSR II, BD Bioscience). The antibodies used in this report include anti-mouse-Igκ-PE (Southern Biotech), anti-human-Igκ-FITC (Southern Biotech), anti-IgM-APC (eBioscience), anti-B220-PerCP-Cy5.5 (Biolegend), anti-CD43-PE (Biolegend), anti-IgD-FITC (eBioscience). Flow cytometry output was analyzed using Flowing Software v2.5.0 (Turku Centre for Biotechnology).

### Analysis of DNA methylation by Southern hybridization

Cellular genomic DNA (5–15 μg) was digested with the specified enzymes, electrophoresed in native (Tris–acetate) agarose gels, denatured and transferred to nitrocellulose. DNA was then hybridized with the specific radioactive probes and analyzed by autoradiography (37). Hybridization was carried out at 65°C for 16 h. The degree of methylation was measured semiquantitatively using a PhosphorImager BAS-1800 (Fuji) and Tina2.10 g software (IsotopenMedgerate GmbH).

### Nuclear extract preparation

Cells (3–5 × 10^6^) were washed in PBS, resuspended in low salt buffer (10 mM HEPES pH 7.9, 10 mM KCl, 0.1 mM EDTA, 0.1 mM EGTA, 1 mM DTT, 0.5 mM PMSF, 20 μg/ml aprotinin, 10 μg/ml leupeptin) and incubated for 10 min on ice. NP-40 was then added to a final concentration of 0.66%, the mixture was vortexed briefly and centrifuged for 30 s, 16,000 *g*. Nuclei were resuspended in high salt buffer (20 mM HEPES pH 7.9, 0.4 mM KCl, 1 mM EDTA, 1 mM EGTA, 1 mM DTT, 1 mM PMSF, 20 μg/ml aprotinin, 10 μg/ml leupeptin) and rotated for 20 min at 4°C. Nuclear debris was removed by centrifugation at 16,000 *g* for 20 min at 4°C.

### Electrophoretic mobility shift assay

Oligonucleotide probes were end-labeled with α^32^P-dCTP using Klenow fragment. Two micrograms of nuclear extract was incubated with 0.3 ng of the radioactive double strand probe in a solution containing 2 μg poly-dI-dC, 10 mM Tris–HCl pH 7.9, 10% glycerol, 100 mM KCl, and 4 mM DTT for 20 min at 25°C. In competition assay, 100-fold molar excess of an unlabeled probe was preincubated for 10 min prior to the addition of the radiolabeled probe. In supershift assays, the indicated antisera (antibodies A1 and A2 kindly provided by Meinrad Busslinger) were added to the nuclear extract 15 min prior to the addition of the probe. Samples were then electrophoresed at room temperature on a 4% polyacrylamide gel (19:1 acrylamide/bis) in 0.25× TBE buffer. Gels were dried and bands were visualized by autoradiography. Probes used for assays were Dm-70 bp 5′-CGATTGTAATTTTATATCGCCAGCAATGGACTGAAACGGTCCGCAACCTCTTCTTTACAACTGGGTGAC-3′ and the Pax5-binding site from the promoter of sea urchin H2a-2.2 5′-GGGTTGTGACGCAGCGGTGGGTGACGACTCCAGAGTCGACA-3′.

### DNAse I footprinting

*Taq*I–*Sac*II fragment (130 bp) from the Dm segment, encompassing the detected Pax5-binding site, was labeled with ^32^P-dCTP at *Taq*I end by a fill-in reaction with Klenow fragment to a specific activity greater than 10^4^ cpm/ng of DNA. Probes were incubated for 20 min at room temperature with 20 μg of nuclear extract in a 50-μl reaction mixture containing 10 mM Tris pH 7.8, 14% glycerol, 57 mM KCl, 4 mM DTT, and 0.2 μg poly(dI-dC). DNase I (0.5–1 U; Promega) diluted in 50 mM MgCl_2_, 10 mM CaCl_2_ was added for 1 min. The reaction was terminated by addition of 150 μl of a stop solution containing 200 mM NaCl, 20 mM EDTA, 1% SDS, and 5 μg yeast tRNA. DNA was extracted with phenol–chloroform, ethanol precipitated, dissolved in loading buffer (deionized Formamide – 5 mM EDTA), denatured for 10 min at 85°C and separated on a 6% polyacrylamide sequencing gel containing 7 M urea. Sequencing reactions performed using the Maxam and Gilbert procedure were run parallel to each probe.

### Bisulfite sequencing

DNA was converted by bisulfite treatment using the EpiTect Bisulfite kit (Qiagen) and amplified by PCR with GoTaq (Promega) using the following primers; BisDm F 5′-TTGATAGATAGTTTAAGGGGTTTTT-3′, BisDm R 5′-ATCTATCACATCTCTATTCTCTTCAAATTA-3′, BisJκ2 F 5′-TTTTTGGAGAATGAATGTTAGTGTAATAAT-3′, BisJκ2 R 5′-TAAAACAATTTTCCCTCCTTAACAC-3′; ionJκ2 F 5′-(ion torrent A adapter)-(index)-GAAATGTTTAAAGAAGTAGGGTAGTTTGT-3′; ionJκ2 R 5′-(ion torrent P1 adapter)-CCCTCCTTAACACCTAATCTAAAAATAA-3′; ionJκ4 F 5′-(ion torrent A adapter)-(index)-ACCAAAAATAACTCATTTAACCAAAATAT-3′, ionJκ4R 5′-(ion torrent P1 adapter)-TGATTTTATGTTAGATTTGTGGGAR-3′. Amplicons were visualized on a 1.5% agarose gel, excised, and purified with the Qiaquick gel extraction kit (Qiagen). Amplicons intended for standard Sanger sequencing were TA cloned using pGEM-T easy kit (Promega). PCR with universal T7 and SP6 primers was performed on transformed colonies and correctly inserted clonal amplicons were sequenced by Sanger sequencing (ABI-Prism-3700). Samples amplified with ion torrent fusion primers were sequenced on an Ion Torrent Personal Genome machine (Invitrogen).

### Chromatin immunoprecipitation

IL-7-dependent pre-B cell cultures were made from the bone marrow of Igκ^WT/ΔDm^ mice as has been previously described (35). Cells were crosslinked with formaldehyde, chromatin extracted, and immunoprecipitated with an antibody directed against Pax5 (5 μg per 30 μg DNA) (SantaCruz). Semi-quantitative PCR was carried out on input DNA compared to immunoprecipitated DNA using primers specific for the Dm element and primers spanning the Dm deletion in order to test the enrichment on the WT and ΔDm alleles separately. PCR amplicons were visualized on an 8% polyacrylamide gel. Primers used: ΔDmChIP-F 5′-CCAAGAGATTGGATCGGAGA-3′, ΔDmChIP-R 5′-CCATGACTTTTGCTGGCTGT-3′; WTDmChIP-F 5′-GGCCACGGTTTTGTAAGACA-3′, WTDmChIP–R 5′-CAGGGTGAACGCCAAATG-3′, CD19-F 5′-GATTTGGAAGAGTGCCTACA-3′, CD19-R 5′-GCCTGCCTCCTACTAAGGTA-3′, β-actin-F 5′-CGCCATGGATGACGATATCG-3′, β-actin-R 5′-CGAAGCCGGCTTTGCACATG-3′.

### Somatic hypermutation analysis of Peyer’s patches B cells

Peyer’s patches (PP) were dissected from the small intestines of 4–6-month-old Igκ^WT/WT^, Igκ^ΔDm/ΔDm^, or Igκ^WT/ΔDm^ mice. PP from three to four mice were pooled for each experiment. PP were mashed through a 70 μm nylon mesh and washed with PBS to produce single cell suspensions. Cells were washed with PBS-0.5% BSA and labeled with PNA-FITC (Vector Labs) and αB220-PE (BD Bioscience). Germinal center B220^+^/PNA^high^ B cells were sorted (FACSStar BD) to greater than 90% purity. WT and ΔDm rearranged Igκ alleles were amplified with Vκ-Degenerate 5′-GTCCCTGCCAGGTTYAGTGGCAGTGGRTCWRGGAC-3′ and R3-1 5′-CAGACCCTGGTCTAATGGTTTGTAACCACATGGG-3′ primers using high fidelity PCR kit (Roche) with an initial denaturation of 4 min at 94°C, followed by 35 cycles of denaturation at 94°C for 15 s and annealing combined with elongation at 68°C for 2 min. 3′ A-overhang nucleotides were added by 20 min incubation with Taq polymerase and ATP at 72°C. PCR fragments corresponding to Vκ–Jκ5 rearrangement of the WT and ΔDM (2.2 and 1.3 kb, respectively) were visualized on a 0.8% agarose gel, excised and purified with the QIAquick gel extraction kit and cloned into the TOPO-2.1 TA cloning vector (Invitrogen). Plasmids from single colonies were prepared and sequenced by Sanger sequencing (ABI-Prism-3700). Sequences were aligned to the Igκ locus and mutations in the 188 bp region downstream of the Vκ–Jκ5 joint were analyzed.

### Pyrosequencing

RNA was extracted using tri-reagent (Sigma-Aldrich) from CD19^+^ MACS sorted (Miltenyi Biotec) splenic cells of BALB/c/Cast Igκ^WT/WT^ and BALB/c/Cast Igκ ^ΔDm/WT^ littermates, as well as control BALB/c and Cast mice. cDNA was prepared with mMLV reverse transcriptase (Promega) using random hexamer primers (Thermo Scientific). Rearranged Igκ transcripts were amplified with Vκ-degenerate: 5′-GTCCCTGCCAGGTTYAGTGGCAGTGGRTCWRGGAC-3′ and biotinylated CκR-5′-GGGAAGCCTCCAAGACCTTA-3′. Resulting amplicons were visualized on a 1.5% agarose gel, excised and purified with the QIAquick gel extraction kit (Qiagen). Allelic distribution of BALB/c/Cast transcripts was assessed by pyrosequencing on a PyroMark Q24 instrument (Qiagen) using Cκ-pyro primer 5′-ACATCAACTTCACCCAT-3′.

### Luciferase reporter assay

M12 cells were transiently transfected using the DEAE dextran method (36) with a luciferase reporter plasmid containing the minimal β-globin promoter TATA box (pTATA), without any additional regulatory elements or with insertions of the Dm element, iEκ, or four NF-κB binding sites immediately upstream of the promoter. The cells were co-transfected with pβ-GAL to normalize for transfection efficiency. Luciferase activity was measured using the Luciferase Assay System (Promega) according to the manufacturer’s instructions.

## Results

### Characterization of the Dm element

**Figure 1:**
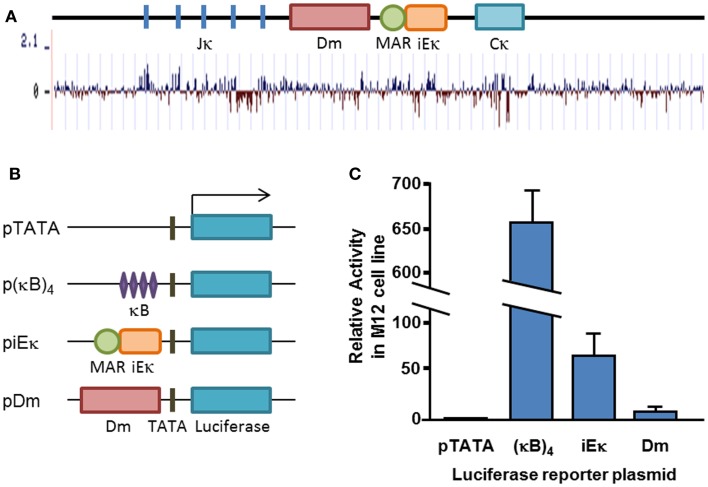
**Conservation and transcriptional activity of the Dm element**. **(A)** Schematic map of the Igκ locus, drawn to scale. Basewise conservation across mammalian genomes is shown graphically, adapted from UCSC genome browser “Conservation” track. **(B)** Schematic map of transfected plasmid constructs. **(C)** Average relative luciferase activity in M12 cells transfected with the indicated plasmids. Plasmids were co-transfected with a constitutive β-Gal-expressing plasmid and luciferase activity was normalized for transfection efficiency to β-Gal activity. Transfection and luciferase assay was carried out at least three times for each construct. Error bars represent the standard deviation of the luciferase activity.

We have previously identified an element lying ~700 bp upstream of the iEκ which facilitates demethylation of the Igκ locus in cell culture, in cooperation with iEκ (32). The element, designated Dm, is not part of the previously defined core iEκ (Figure 1A). The Dm element, as determined by our previous experiments, spans ~1 kb and contains numerous areas which are conserved throughout different species (Figure 1A). The element itself contains a stretch of ~200 bp with the highest density of CpG sites found within the Igκ locus. In order to see whether this element was transcriptionally active, we tested its functionality in an enhancer reporter assay. We compared its activity in a reporter plasmid to the well-characterized iEκ (Figure 1B). Luciferase assays show that the Dm element acts only as a weak transcriptional enhancer which is about sevenfold weaker than the core intronic enhancer in M12 B cell lymphoma cells (Figure 1C), suggesting that the Dm element on its own does not exert its effect by direct transcriptional activation.

### Pax5 binding at the Dm element

**Figure 2:**
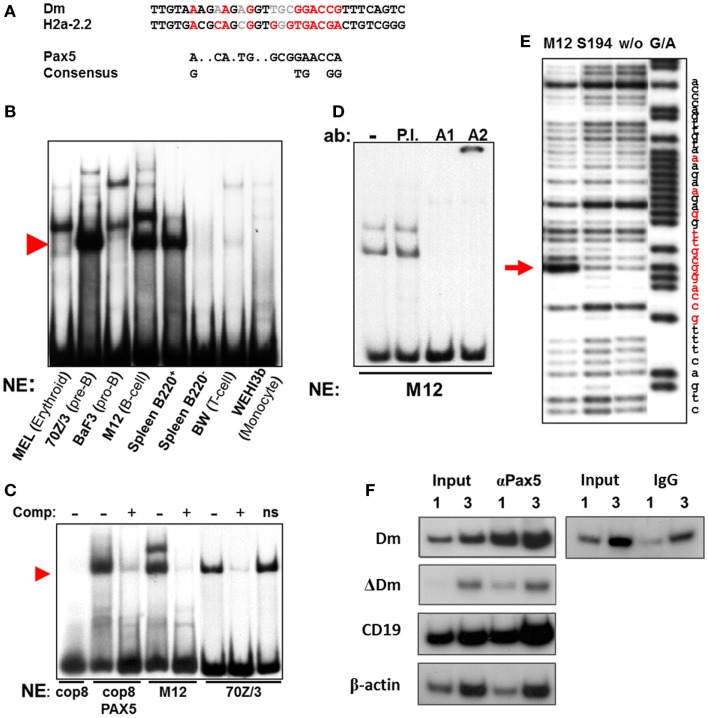
**Pax5-binding within the Dm element**. **(A)** Comparison of the putative Pax5-binding site within the Dm element, known Pax5-binding site in sea urchin H2a-2.2 promoter and Pax5 consensus sequence. Bases which match the consensus sequence are marked in red, while bases which do not match are marked in gray. **(B)** Electro-mobility shift assay (EMSA) of *Taq*I–*Bst*EII 70 bp fragment from the Dm element, containing the putative Pax5-binding site, with the indicated hematopoietic cell type nuclear extracts. Red arrowhead indicates B cell lineage-specific shift. **(C)** Competition EMSA. Unlabeled H2a-2.2 probe competes with radioactive *Taq*I–*Bst*EII Dm probe and reduces shift in B lineage cell extracts (M12, 70Z/3), and in extracts of fibroblast cells (cop8) transfected with a Pax5 expression vector. Nonsense unlabeled probe (ns) is unable to compete with Dm probe. Red arrowhead indicates specific shift. **(D)** Supershift assay with *Taq*I–*Bst*EII Dm probe, which was incubated with antibodies (ab) raised against the DNA-binding site of Pax5 (A1), the transactivation domain (A2), or general rabbit antisera (P.I.). **(E)** DNase I footprinting assay on end-labeled *Taq*I–*Sac*II probe from the Dm element. Labeled probe was incubated with nuclear extracts from the indicated cell types. A control Maxam and Gilbert reaction (G/A) was run in parallel. Footprint specific to Pax5-expressing M12 cells is indicated with a red arrow. Location of the putative Pax5-binding site is marked in red on the nucleotide sequence. **(F)** Chromatin immunoprecipitation (ChIP) enrichment of Pax5 at the Dm element in Igκ^wt/ΔDm^ cultured pre-B cells. Enrichment was measured by semi-quantitative PCR *via* comparison of the input DNA (Input) to the immunoprecipitated fraction (αPax5), using primers specific to the Dm element (Dm) and the deleted allele (ΔDm). One and three times the amount of PCR template were run in parallel to ensure linearity. Positive (CD19 promoter) and negative (β-actin promoter) controls for Pax5-binding were analyzed in parallel to ensure specificity of the enrichment. ChIP with a non-specific antibody (IgG) does not enrich the Dm element.

*Cis* regulatory elements, such as enhancers and promoters, convey their influence on cellular phenotypes by binding *trans* regulatory transcription factors, which mediate transcription and changes in chromatin structure. As the Igκ locus is selectively active in B cells, starting from the pre-B cell stage, we speculated that the Dm element may bind B cell-specific transcription factors, thus mediating the changes it induces. Upon searching for potential binding sites for key B cell transcription regulators, we identified an area within the CpG-rich segment with remarkable similarity to the Pax5 consensus sequence (38) (Figure 2A). A 70-bp probe containing this sequence is shifted to a specific height when incubated with nuclear extracts from B lineage cells which have passed the pro-B cell stage, but not in other cell types tested in an electro-mobility shift assay (EMSA) (Figure 2B). These results clearly show that the binding of this protein is specific for the stages when the Igκ locus is active. Notably, this specific shift can be attained using a fibroblast extract, which normally does not produce such a shift, by forced expression of Pax5 (Figure 2C), and titrated away by competition with a probe containing the Pax5-binding site of the H2a-2.2 promoter, strongly implying that indeed the Pax5 protein is binding at this site. When the nuclear extract is incubated with an antibody raised against the DNA-binding domain of Pax5 (designated A1), the shift on the EMSA gel disappears, whereas incubation with an antibody recognizing the Pax5 transactivation domain (designated A2) introduces a supershift, confirming that the 70-bp probe indeed specifically binds the Pax5 transcription factor (Figure 2D). DNase I footprinting using nuclei of the Pax5-expressing M12 B cell lymphoma cell line shows a definitive protection at the putative Pax5-binding site in comparison to S194 plasmacytoma cells which do not express Pax5 (Figure 2E). Interestingly, this specific footprint correlates precisely with the predicted Pax5-binding site. ChIP was performed on pre-B cells from Igκ^WT/ΔDm^ mice (introduction of the ΔDm allele into mice is described in Section Characterization of Methylation, Rearrangement and B Cell Development in Dm Knockout Mice) with an antibody recognizing the Pax5 protein. While the Dm positive allele showed significant enrichment for Pax5, the deleted allele was not enriched for Pax5-binding (Figure 2F). These results indicate that Pax5 indeed binds in this region *in vivo* and that the binding is directly dependent on the presence of the Dm element. Altogether, the above described data shows that Pax5 specifically binds to the Dm element *in vivo*.

### Dm facilitates DNA demethylation of Igκ transgenes

**Figure 3:**
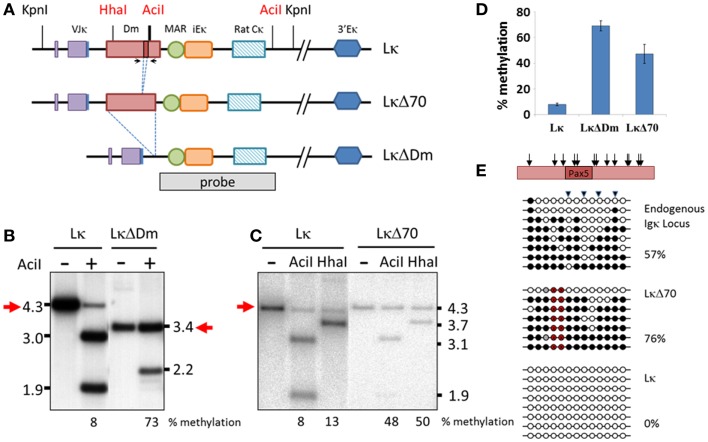
**Effect of Dm element on methylation of Igκ locus in transgenic mice**. **(A)** Schematic map of the transgenes and probe used in experiments. Bold *Aci*I line indicates multiple *Aci*I restriction sites within close proximity. **(B,C)** Southern blot assay assessing methylation state of Lκ, LκΔDm **(B)** and LκΔ70 **(C)** transgenes in B220^+^ splenic B cells. DNA was digested with *Kpn*I either alone or with methylation sensitive enzymes *Aci*I or *Hha*I and hybridized with the indicated probe. Methylation levels were quantified by phosphorImager. **(D)** Graphical representation of the average methylation levels at *Aci*I sites of the indicated transgenes, as quantified by phosphorImager. Methylation was measured in DNA from two to four independent founder mice for each transgene. Error bars represent standard deviation of the methylation levels. **(E)** Bisulfite analysis of CpGs flanking the Pax5-binding sequence of the Dm element. Triangles mark CpGs recognized by *Aci*I restriction enzyme. Black circles signify methylated, white signify unmethylated, gray signify undetermined, and red signify CpGs deleted in transgene. Percentage of methylated CpGs is noted. Relative location of the CpGs within the Dm element is indicated with arrows on schematic representation of the genomic region. Location of primers is marked by arrows in **(A)**.

We wished to further investigate the role of the Dm element in demethylation of the Igκ locus. In order to do so, we introduced a well-characterized transgene (34, 39) containing a pre-rearranged Igκ allele to mice, termed Lκ (Figure 3A). Two additional transgenic mice were produced with modified constructs, one containing a deletion of the entire Dm locus, termed LκΔDm, and the second containing a deletion of the 70 bp region containing the Pax5-binding site, termed LκΔ70 (Figure 3A). DNA from splenic B220^+^ cells was assayed for the methylation of these transgenes by restriction analysis, which allows for simple differentiation between the transgenic and endogenous regions. Digestion with *Kpn*I gave rise to a 4.3-kb fragment in the Lκ and LκΔ70 transgenes and a 3.4-kb fragment in the LκΔDm transgene, whereas the endogenous locus yields a 15-kb fragment. These fragments were further digested with methylation-sensitive restriction enzymes *Aci*I and *Hha*I (*Hha*I was not used to assess the LκΔDm state since the *Hha*I site is deleted in this transgene). The digested DNA was hybridized with a probe recognizing the MAR and iEκ sequences. To assess the level of methylation, the amount of the undigested DNA was measured using a PhosphorImager. Interestingly, while the Lκ transgene was almost completely unmethylated, with only 8% of the DNA remaining undigested (Figures 3B,C), the LκΔDm transgene was highly methylated (73%) (Figures 3B,D), indicating that indeed the Dm element facilitates the hypomethylation of the Igκ locus in B cells. Notably, deletion of only 70 bp from the Dm in the LκΔ70 transgene reduced the ability of the transgene to become unmethylated (50%) (Figures 3C,D).

Bisulfite analysis of the CpG-rich region surrounding the Pax5-binding site in the endogenous locus, Lκ and LκΔ70 transgenes showed a picture that agrees quite nicely with the above results (the LκΔDm was not assayed in this manner, since this region is deleted within the transgene). These results take into consideration the difference between the methylation levels measured by bisulfite sequencing, which probes all CpG sites in the region, and the restriction analysis which measures the methylation only at the sites which correspond to the digestion site. The endogenous locus is close to 50% methylated, as expected from a region which undergoes monoallelic demethylation (Figure 3E). The LκΔ70 transgene is 76% methylated, while the Lκ transgene is completely unmethylated (Figure 3E). In order to see how these results correlate with the restriction analysis, the percent of sequences which would be protected from *Aci*I digestion was assessed. Fifty-seven percent of the LκΔ70 sequences remain protected, supporting the restriction analysis results. These experiments clearly show that the Dm element contributes to the demethylation of the Igκ locus *in vivo*, results that support previously published data obtained from cell culture systems.

### Characterization of methylation, rearrangement, and B cell development in Dm knockout mice

**Figure 4:**
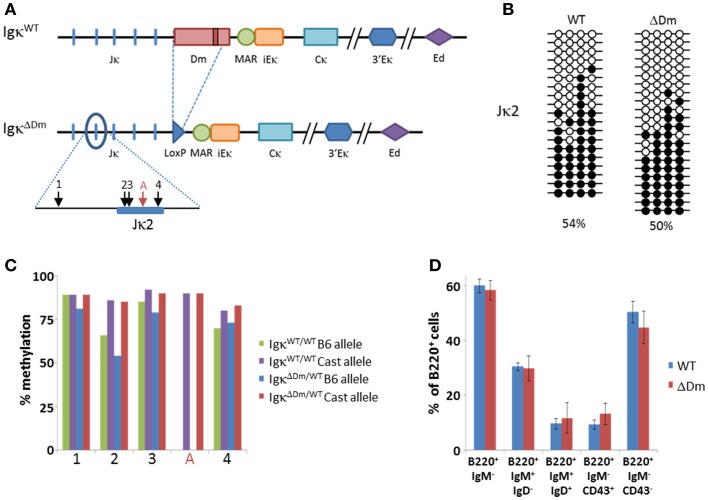
**Effect of deletion of the Dm element at the endogenous locus on Igκ methylation and B cell development in the bone marrow**. **(A)** Schematic map of the endogenous Igκ locus in wild-type (WT) and Dm knockout (ΔDm) mice. Relative locations of CpGs in Jκ2 region are indicated with arrows. CpG present only in Castaneous (Cast) strain is marked with a red arrow. **(B)** Bisulfite analysis of CpGs at the Jκ2 region in splenic CD19^+^ B cells from WT and ΔDm mice. Black circles signify methylated CpGs, white circles signify unmethylated CpGs. Percentage of methylated CpGs is noted. **(C)** Bisulfite analysis by high-throughput sequencing of Jκ2 region from CD19^+^ bone marrow pre-B cells from Rag1^−/−^ C57BL/6/Castaneous IgH-3H9-Tg mice with or without a deletion of the Dm element on the C57BL/6 (B6) allele. Copies (1600–3000) of each CpG from each genotype were analyzed. Alleles were differentiated by strain-specific polymorphic sites within the amplified regions. The methylation state of each CpG is summarized graphically. **(D)** Summary of proportions of B cell populations within bone marrows of WT and ΔDm mice. Error bars mark standard deviation. Six mice were analyzed in each group. Representative FACS plots can be seen in Figure S4 in Supplementary Material.

Given the results in transgenic mice, we generated a knockout mouse in which the entire Dm element in the endogenous locus was replaced with a LoxP-flanked Neo gene which was then excised from the genome (Figure 4A; Figure S1 in Supplementary Material). We assessed the methylation pattern of the Igκ locus by bisulfite analysis of the Jκ2 fragment in *ex vivo* mature B cells. Surprisingly, given the strong phenotype in transgenic mice, no significant difference was seen between the methylation levels of Igκ^WT/WT^ and Igκ^ΔDm/ΔDm^ mice (Figure 4B).

We then proceeded to investigate whether the methylation patterns at the Igκ locus are affected by deletion of the Dm element in the pre-B cell stage, which is the very first stage in which demethylation of the locus is detected. To this end, Igκ^ΔDm^ mice were bred onto a Rag1^−/−^ background, effectively blocking rearrangement of the Igκ locus and differentiation to the mature B cell stage. Expression of a pre-rearranged IgH transgene was ensured in order to allow the cells to express the pre-BCR and differentiate to the pre-B cell stage. These mice were further bred with Rag1^−/−^ *M. castaneous* mice, which contain an intact Dm element, thus allowing distinction between the WT and the Dm-deleted alleles based on the strain-specific DNA polymorphisms. *Ex vivo* CD19^+^ bone marrow cells were purified from B6/Cast Rag1^-/-^ IgH^+^ Igκ^ΔDm/WT^ and B6/Cast Rag1^-/-^ IgH^+^ Igκ^WT/WT^ mice and the methylation of the Jκ2 and Jκ4 segments was determined by high-throughput sequencing. We did not, however, detect significant differences in levels of methylation between the WT and ΔDm alleles (Figure 4C, Figure S2 in Supplementary Material). Taken together, we find that, while the Dm element plays a role in demethylation of the Igκ locus in transgenes, this role is not translated to the endogenous locus, probably due to redundancy of the many enhancers of the locus, not all of which are present in the transgene.

We explored the possibility that the Dm element may affect other developmental processes pertaining the Igκ locus and normal B cell development, as has been observed for *cis* regulatory elements in the locus such as the enhancers. There was no significant difference seen in the levels of rearrangement of the WT versus ΔDm allele, as assessed by FACS and pyrosequencing analyses (Figure S3 in Supplementary Material). The pyrosequencing results also indicate that the level of Igκ transcription is not changed by the deletion of the Dm element, supporting the above described results showing that the Dm element is a weak transcriptional enhancer. The B cell development in the bone marrow of Igκ^ΔDm/ΔDm^ mice appeared normal, with proportions of pro-, pre-, immature, and mature B cells similar to those of WT mice (Figure 4D, Figure S4 in Supplementary Material). Overall, these results indicate that, in the endogenous locus, deletion of the Dm element does not curtail these early stages of B cell development.

### Effect of Dm element on SHM

**Figure 5:**
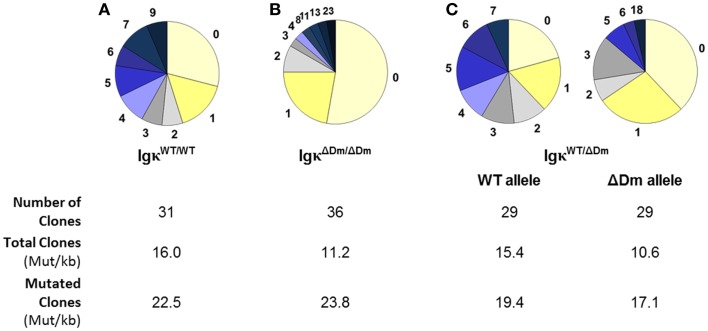
**Effect of deletion of the Dm element at the endogenous locus on somatic hypermutations**. Somatic hypermutations in B220^+^PNA^high^ Peyer’s patches germinal center B cells from **(A)** Igκ^WT/WT^, **(B)** Igκ^ΔDm/ΔDm^, and **(C)** Igκ^WT/ΔDm^ mice. Pie charts indicate the number of mutations sequenced in a 188-bp region immediately downstream of the Vκ–Jκ5 joint. Number of colonies sequenced from each mouse and mutation rate per kilobase in total and mutated clones are indicated.

We investigated whether deletion of the Dm element affects a later stage of Igκ maturation, specifically the process of SHM in activated B cells. Levels of SHM in Igκ^WT/WT^ mice versus Igκ^ΔDm/ΔDm^ mice were examined, and a significant drop in amount of mutations in the germinal center B220^+^PNA^high^ B cells from Peyer’s patches of the Dm negative mice was observed (Figures 5A,B). In order to rule out mouse to mouse variation, which could potentially give rise to such an effect, SHM in heterozygous Igκ^WT/ΔDm^ mice was assessed. Here too, the proportion of mutations on the ΔDm allele was lower than on the WT allele (Figure 5C). As a control, a similar number of colonies were sequenced from B220^+^PNA^low^ cells, with no mutations detected (data not shown). While the average number of mutations is lower in the ΔDm allele, sequences which have undergone SHM do so at an efficiency similar to the WT allele, as seen when examining the mutation rate in total sequences versus rate in mutated sequences (Figure 5), suggesting that the Dm element affects the recruitment but not the processivity of the machinery involved in SHM. These results indicate that the Dm element, which is immediately adjacent to the intronic MAR and iEκ, helps promote SHM. This is particularly notable, as deletion of the MAR/iEκ region on its own has no discernable effect on the normal SHM process (18). Our results clearly show that the Dm element contributes to proper SHM at the Igκ locus, a role which has not been previously attributed to the intronic enhancer region.

## Discussion

In this paper, we characterized a novel *cis* regulatory element situated within the Jκ–Cκ intron of the Igκ locus. This sequence was originally identified as an element which lies adjacent to iEκ and contributes to its demethylating activity, as deletion of either element was sufficient to abolish demethylation in a cell culture system (32). In our present study, we find that the Dm element is necessary for hypomethylation of the Igκ locus of the Lκ transgene *in vivo*, but is dispensable for the demethylation of the endogenous locus. The apparent discrepancy between the phenotype in these two cases may be due to the fact that the transgene contains the sequences in the Igκ locus up to the 3′Eκ, but does not include the Ed. The three Igκ enhancers work cooperatively and, to a certain extent, redundantly to activate and demethylate the locus. Previous studies have shown that deletion of any single enhancer has only a small effect on the developmentally regulated DNA demethylation, whereas the combined lack of two enhancers abolishes the demethylation process (16, 19). Another difference between the transgene and the endogenous locus is that the transgene contains a pre-rearranged Igκ. It is possible that the Dm element only affects the demethylation when the locus is in a rearranged configuration, but not in the germline conformation. In this study, we see that the deletion of the Dm sequence, which is not part of the core iEκ, greatly impedes the demethylation process in the transgene, indicating that the Dm element contributes to the activity of iEκ, possibly as a co-enhancer. As the Dm is only a weak transcriptional enhancer as a solitary element, it is the combined activity with neighboring *cis* acting elements which gives rise to the full activity.

The mechanisms by which genomic loci undergo targeted demethylation have long been shrouded in mystery (40). Findings from recent years have pointed to the Tet family of enzymes as possible catalysts of the demethylation process, *via* oxidation of the methyl group into a hydroxymethyl moiety (41). When acting as a demethylation intermediate, the hydroxymethylated cytosine is then either passively diluted during DNA replication (42), as it is not recognized by the methylation maintenance machinery (43, 44), or, conversely, is actively excised from the genome and replaced with an unmethylated nucleotide (45, 46). Targeting of Tet proteins to specific genomic loci is sufficient to induce local demethylation (47, 48). Tet2 has been implicated in the active demethylation of tissue-specific genes in postmitotic human monocytes (49). Additionally, Tet2 has been found to bind PU.1 (50) and EBF1 (51) in the hematopoietic system. A recent report has uncovered a different strategy to induce demethylation by which DNMT1, the maintenance DNA methyltransferase, is sequestered from specific genomic loci by binding non-coding RNA. This prevents the placement of methyl groups on DNA during replication and, in turn, brings about passive demethylation of a defined region (52). It is still unclear what mechanism is implemented by the *cis* regulatory elements to demethylate the Igκ locus during B cell development, especially since deletion of Tet2, the strongest Tet candidate in the immune system, causes leukemia in mice (53–55), which masks many of the tissue-specific effects that may occur as a result. As methylation is a strong barrier to the rearrangement process (8), future studies can address this issue.

We have identified a sequence within the Dm element which binds the B cell lineage specifier Pax5. This site is bound by Pax5 starting with the pre-B cell stage, up to mature B cells, but is unbound in Baf3 pro-B cells, where the Igκ locus is not yet activated and made accessible for rearrangement, nor in plasma cells where Pax5 expression is down-regulated. It should be noted, though, that the Baf3 pro-B cell line tested here does not express Pax5 (56), whereas most pro-B cells do, and as such we are unable to rule out the possibility that Pax5 is already bound at the pro-B cell stage. This is the first report, to our knowledge, of a Pax5-binding site within the Igκ locus which binds Pax5 at the time of locus activation. Previous reports have located sites in 3′Eκ (24) and in the K-I–K-II (29, 30) regulatory elements in which Pax5 plays a repressive role and where binding is lost upon Igκ locus activation. The new site we report is particularly interesting, considering that Pax5 is known to be directly necessary for Igκ locus activation and κ_0_ germline transcription in pre-B cells (31). We find that Pax5-binding in the vicinity of the Jκ–Cκ intron is dependent on the presence of the Dm element and that the Pax5-binding site contributes to the demethylating capabilities of the Dm element. While this clearly cannot be the only Pax5-binding site, since ΔDm pre-B cells maintain their full ability to rearrange the Igκ locus, this site highlights the potency of this B cell identity protein in one more area of B cell development.

It should be noted that the sequence of the Pax5-binding site within the Dm element is conserved among rodents, but not in the human-Igκ locus, though other aspects, such as the CpG-dense region, are. This is not the only aspect which differs between the human and murine counterparts of the Igκ locus. For example, the Sis element, a transcriptional silencer which has been shown to recruit the Igκ locus to the pericentromeric heterochromatin in mice, is not conserved in the human locus (57). It stands to reason that regulation of the human and murine Igκ loci may differ somewhat, as the strongly biased usage of the κ versus λ chain seen in mice (where 95% of mature B cells express the κ chain) is not present in humans, which have a ratio of 60:40 of κ versus λ usage (58). This could be due to differences in the RR of the human and murine Igκ locus that may contribute to this phenomenon.

While the deletion of the Dm element did not, on its own, affect the methylation status of the endogenous Igκ locus, nor the relative amount of the deleted allele which underwent rearrangement, we observed a decrease in the levels of SHM on Igκ alleles lacking the Dm element. The role of the Dm element in facilitating SHM appears to be independent of the iEκ/MAR region, since the combined deletion of the iEκ and MAR elements has no perceptible effect on SHM (18). The lower level of SHM does not appear to be the result of lower levels of Igκ transcription, since deletion of the Dm element does not lower the levels of Igκ RNA observed in mature B cells. Deletion of the Dm element appears to cause inefficient recruitment of the mutating machinery, but once the machinery is in place, the mutation efficiency is similar to the WT locus. The element may therefore function by efficiently recruiting the mutation machinery to the locus, possibly by key regulators such as Pax5 which are bound to the Dm element. Pax5 itself has a known role in SHM by activating the transcription of the *Aicda* gene, encoding the AID protein, which is the deaminase responsible for SHM (59, 60). It may be that Pax5 plays more than one role in SHM induction. The role of the Dm element in SHM fits in well with its location, which is almost immediately adjacent to the Vκ–Jκ rearranged region which is the hotspot for SHM.

In conclusion, we have characterized the Dm sequence as an element that regulates the Igκ locus during different stages of B cell development. The Dm is both a team player, cooperating with the three characterized enhancers to demethylate the locus for rearrangement, as well as an element that affects the locus in its own right in allowing efficient SHM. This report adds to our understanding of the complex regulation of the Igκ locus, which undergoes many drastic changes during development and must be fine-tuned for each developmental stage.

## Author Contributions

Rena Levin-Klein, Andrei Kirillov, and Chaggai Rosenbluh designed the experiments, did the research, and interpreted the results. Howard Cedar and Yehudit Bergman directed the study. Rena Levin-Klein and Yehudit Bergman wrote the manuscript.

## Conflict of Interest Statement

The authors declare that the research was conducted in the absence of any commercial or financial relationships that could be construed as a potential conflict of interest.

## Supplementary Material

The Supplementary Material for this article can be found online at http://www.frontiersin.org/journal/10.3389/fimmu.2014.00240/abstract

## References

[1] Gellert M V(D)J recombination: RAG proteins, repair factors, and regulation. Annu Rev Biochem (2002) 71:101–32 10.1146/annurev.biochem.71.090501.150203 12045092

[2] Yancopoulos GD Alt FW Developmentally controlled and tissue-specific expression of unrearranged VH gene segments. Cell (1985) 40(2):271–81 10.1016/0092-8674(85)90141-2 2578321

[3] Constantinescu A Schlissel MS Changes in locus-specific V(D)J recombinase activity induced by immunoglobulin gene products during B cell development. J Exp Med (1997) 185(4):609–20 10.1084/jem.185.4.609 9034140 PMC2196138

[4] Stanhope-Baker P Hudson KM Shaffer AL Constantinescu A Schlissel MS Cell type-specific chromatin structure determines the targeting of V(D)J recombinase activity in vitro. Cell (1996) 85(6):887–97 10.1016/S0092-8674(00)81272-6 8681383

[5] Mostoslavsky R Singh N Kirillov A Pelanda R Cedar H Chess A Kappa chain monoallelic demethylation and the establishment of allelic exclusion. Genes Dev (1998) 12(12):1801–11 10.1101/gad.12.12.1801 9637682 PMC316908

[6] Nakase H Takahama Y Akamatsu Y Effect of CpG methylation on RAG1/RAG2 reactivity: implications of direct and indirect mechanisms for controlling V(D)J cleavage. EMBO Rep (2003) 4(8):774–80 10.1038/sj.embor.embor904 12897800 PMC1326344

[7] Goldmit M Ji Y Skok J Roldan E Jung S Cedar H Epigenetic ontogeny of the Igk locus during B cell development. Nat Immunol (2005) 6(2):198–203 10.1038/ni1154 15619624

[8] Goldmit M Schlissel M Cedar H Bergman Y Differential accessibility at the kappa chain locus plays a role in allelic exclusion. EMBO J (2002) 21(19):5255–61 10.1093/emboj/cdf518 12356741 PMC129040

[9] Odegard VH Schatz DG Targeting of somatic hypermutation. Nat Rev Immunol (2006) 6(8):573–83 10.1038/nri1896 16868548

[10] Fraenkel S Mostoslavsky R Novobrantseva TI Pelanda R Chaudhuri J Esposito G Allelic ‘choice’ governs somatic hypermutation in vivo at the immunoglobulin kappa-chain locus. Nat Immunol (2007) 8(7):715–22 10.1038/ni1476 17546032

[11] Queen C Baltimore D Immunoglobulin gene transcription is activated by downstream sequence elements. Cell (1983) 33(3):741–8 10.1016/0092-8674(83)90016-8 6409419

[12] Meyer KB Neuberger MS The immunoglobulin kappa locus contains a second, stronger B-cell-specific enhancer which is located downstream of the constant region. EMBO J (1989) 8(7):1959–64 2507312 10.1002/j.1460-2075.1989.tb03601.x PMC401057

[13] Liu ZM George-Raizen JB Li S Meyers KC Chang MY Garrard WT Chromatin structural analyses of the mouse Igkappa gene locus reveal new hypersensitive sites specifying a transcriptional silencer and enhancer. J Biol Chem (2002) 277(36):32640–9 10.1074/jbc.M204065200 12080064

[14] Xu Y Davidson L Alt FW Baltimore D Deletion of the Ig kappa light chain intronic enhancer/matrix attachment region impairs but does not abolish V kappa J kappa rearrangement. Immunity (1996) 4(4):377–85 10.1016/S1074-7613(00)80251-4 8612132

[15] Gorman JR van der Stoep N Monroe R Cogne M Davidson L Alt FW The Ig(kappa) enhancer influences the ratio of Ig(kappa) versus Ig(lambda) B lymphocytes. Immunity (1996) 5(3):241–52 10.1016/S1074-7613(00)80319-2 8808679

[16] Inlay M Alt FW Baltimore D Xu Y Essential roles of the kappa light chain intronic enhancer and 3′ enhancer in kappa rearrangement and demethylation. Nat Immunol (2002) 3(5):463–8 10.1038/ni790 11967540

[17] Xiang Y Garrard WT The Downstream Transcriptional Enhancer, Ed, positively regulates mouse Ig kappa gene expression and somatic hypermutation. J Immunol (2008) 180(10):6725–32 10.4049/jimmunol.180.10.6725 18453592 PMC2424255

[18] Inlay MA Gao HH Odegard VH Lin T Schatz DG Xu Y Roles of the Ig kappa light chain intronic and 3′ enhancers in Igk somatic hypermutation. J Immunol (2006) 177(2):1146–51 10.4049/jimmunol.177.2.1146 16818772

[19] Zhou X Xiang Y Garrard WT The Igkappa gene enhancers, E3′ and Ed, are essential for triggering transcription. J Immunol (2010) 185(12):7544–52 10.4049/jimmunol.1002665 21076060 PMC3059262

[20] Inlay MA Lin T Gao HH Xu Y Critical roles of the immunoglobulin intronic enhancers in maintaining the sequential rearrangement of IgH and Igk loci. J Exp Med (2006) 203(7):1721–32 10.1084/jem.20052310 16785310 PMC2118354

[21] Zhou X Xiang Y Ding X Garrard WT A new hypersensitive site, HS10, and the enhancers, E3′and Ed, differentially regulate Igkappa gene expression. J Immunol (2012) 188(6):2722–32 10.4049/jimmunol.1102758 22323542 PMC3294001

[22] Cockerill PN Garrard WT Chromosomal loop anchorage of the kappa immunoglobulin gene occurs next to the enhancer in a region containing topoisomerase II sites. Cell (1986) 44(2):273–82 10.1016/0092-8674(86)90761-0 3002631

[23] McDevit DC Perkins L Atchison ML Nikolajczyk BS The Ig kappa 3′ enhancer is activated by gradients of chromatin accessibility and protein association. J Immunol (2005) 174(5):2834–42 10.4049/jimmunol.174.5.2834 15728493

[24] Shaffer AL Peng A Schlissel MS In vivo occupancy of the kappa light chain enhancers in primary pro- and pre-B cells: a model for kappa locus activation. Immunity (1997) 6(2):131–43 10.1016/S1074-7613(00)80420-3 9047235

[25] Roque MC Smith PA Blasquez VC A developmentally modulated chromatin structure at the mouse immunoglobulin kappa 3′ enhancer. Mol Cell Biol (1996) 16(6):3138–55 8649425 10.1128/mcb.16.6.3138 PMC231308

[26] Lazorchak AS Schlissel MS Zhuang Y E2A and IRF-4/Pip promote chromatin modification and transcription of the immunoglobulin kappa locus in pre-B cells. Mol Cell Biol (2006) 26(3):810–21 10.1128/MCB.26.3.810-821.2006 16428437 PMC1347029

[27] Inlay MA Tian H Lin T Xu Y Important roles for E protein binding sites within the immunoglobulin kappa chain intronic enhancer in activating Vkappa Jkappa rearrangement. J Exp Med (2004) 200(9):1205–11 10.1084/jem.20041135 15504821 PMC2211861

[28] Maitra S Atchison M BSAP can repress enhancer activity by targeting PU.1 function. Mol Cell Biol (2000) 20(6):1911–22 10.1128/MCB.20.6.1911-1922.2000 10688639 PMC110809

[29] Sato H Wang D Kudo A Dissociation of Pax-5 from KI and KII sites during kappa-chain gene rearrangement correlates with its association with the underphosphorylated form of retinoblastoma. J Immunol (2001) 166(11):6704–10 10.4049/jimmunol.166.11.6704 11359826

[30] Tian J Okabe T Miyazaki T Takeshita S Kudo A Pax-5 is identical to EBB-1/KLP and binds to the VpreB and lambda5 promoters as well as the KI and KII sites upstream of the Jkappa genes. Eur J Immunol (1997) 27(3):750–5 10.1002/eji.1830270325 9079818

[31] Sato H Saito-Ohara F Inazawa J Kudo A Pax-5 is essential for kappa sterile transcription during Ig kappa chain gene rearrangement. J Immunol (2004) 172(8):4858–65 10.4049/jimmunol.172.8.4858 15067064

[32] Lichtenstein M Keini G Cedar H Bergman Y B cell-specific demethylation: a novel role for the intronic kappa chain enhancer sequence. Cell (1994) 76(5):913–23 10.1016/0092-8674(94)90365-4 8124725

[33] Casellas R Shih TA Kleinewietfeld M Rakonjac J Nemazee D Rajewsky K Contribution of receptor editing to the antibody repertoire. Science (2001) 291(5508):1541–4 10.1126/science.1056600 11222858

[34] Meyer KB Sharpe MJ Surani MA Neuberger MS The importance of the 3′-enhancer region in immunoglobulin kappa gene expression. Nucleic Acids Res (1990) 18(19):5609–15 10.1093/nar/18.19.5609 2120679 PMC332290

[35] Farago M Rosenbluh C Tevlin M Fraenkel S Schlesinger S Masika H Clonal allelic predetermination of immunoglobulin-kappa rearrangement. Nature (2012) 490(7421):561–5 10.1038/nature11496 23023124

[36] Gulick T Transfection using DEAE-dextran. Curr Protoc Cell Biol (2003) Chapter 20:Unit20.4. 10.1002/0471143030.cb2004s19 18228428

[37] Southern EM Detection of specific sequences among DNA fragments separated by gel electrophoresis. J Mol Biol (1975) 98(3):503–17 10.1016/S0022-2836(75)80083-0 1195397

[38] Czerny T Schaffner G Busslinger M DNA sequence recognition by Pax proteins: bipartite structure of the paired domain and its binding site. Genes Dev (1993) 7(10):2048–61 10.1101/gad.7.10.2048 8406007

[39] Sharpe MJ Milstein C Jarvis JM Neuberger MS Somatic hypermutation of immunoglobulin kappa may depend on sequences 3′ of C kappa and occurs on passenger transgenes. EMBO J (1991) 10(8):2139–45 1905999 10.1002/j.1460-2075.1991.tb07748.x PMC452901

[40] Ooi SK Bestor TH The colorful history of active DNA demethylation. Cell (2008) 133(7):1145–8 10.1016/j.cell.2008.06.009 18585349

[41] Tahiliani M Koh KP Shen Y Pastor WA Bandukwala H Brudno Y Conversion of 5-methylcytosine to 5-hydroxymethylcytosine in mammalian DNA by MLL partner TET1. Science (2009) 324(5929):930–5 10.1126/science.1170116 19372391 PMC2715015

[42] Inoue A Zhang Y Replication-dependent loss of 5-hydroxymethylcytosine in mouse preimplantation embryos. Science (2011) 334(6053):194 10.1126/science.1212483 21940858 PMC3799877

[43] Hashimoto H Liu Y Upadhyay AK Chang Y Howerton SB Vertino PM Recognition and potential mechanisms for replication and erasure of cytosine hydroxymethylation. Nucleic Acids Res (2012) 40(11):4841–9 10.1093/nar/gks155 22362737 PMC3367191

[44] Otani J Kimura H Sharif J Endo TA Mishima Y Kawakami T Cell cycle-dependent turnover of 5-hydroxymethyl cytosine in mouse embryonic stem cells. PLoS One (2013) 8(12):e82961 10.1371/journal.pone.0082961 24340069 PMC3858372

[45] Guo JU Su Y Zhong C Ming GL Song H Hydroxylation of 5-methylcytosine by TET1 promotes active DNA demethylation in the adult brain. Cell (2011) 145(3):423–34 10.1016/j.cell.2011.03.022 21496894 PMC3088758

[46] He YF Li BZ Li Z Liu P Wang Y Tang Q Tet-mediated formation of 5-carboxylcytosine and its excision by TDG in mammalian DNA. Science (2011) 333(6047):1303–7 10.1126/science.1210944 21817016 PMC3462231

[47] Maeder ML Angstman JF Richardson ME Linder SJ Cascio VM Tsai SQ Targeted DNA demethylation and activation of endogenous genes using programmable TALE-TET1 fusion proteins. Nat Biotechnol (2013) 31(12):1137–42 10.1038/nbt.2726 24108092 PMC3858462

[48] Chen H Kazemier HG de Groote ML Ruiters MH Xu GL Rots MG Induced DNA demethylation by targeting ten-eleven translocation 2 to the human ICAM-1 promoter. Nucleic Acids Res (2014) 42(3):1563–74 10.1093/nar/gkt1019 24194590 PMC3919596

[49] Klug M Schmidhofer S Gebhard C Andreesen R Rehli M 5-Hydroxymethylcytosine is an essential intermediate of active DNA demethylation processes in primary human monocytes. Genome Biol (2013) 14(5):R46 10.1186/gb-2013-14-5-r46 23705593 PMC4053946

[50] de la Rica L Rodriguez-Ubreva J Garcia M Islam AB Urquiza JM Hernando H PU.1 target genes undergo Tet2-coupled demethylation and DNMT3b-mediated methylation in monocyte-to-osteoclast differentiation. Genome Biol (2013) 14(9):R99 10.1186/gb-2013-14-9-r99 24028770 PMC4054781

[51] Guilhamon P Eskandarpour M Halai D Wilson GA Feber A Teschendorff AE Meta-analysis of IDH-mutant cancers identifies EBF1 as an interaction partner for TET2. Nat Commun (2013) 4:2166 10.1038/ncomms3166 23863747 PMC3759038

[52] Di Ruscio A Ebralidze AK Benoukraf T Amabile G Goff LA Terragni J DNMT1-interacting RNAs block gene-specific DNA methylation. Nature (2013) 503(7476):371–6 10.1038/nature12598 24107992 PMC3870304

[53] Ko M Bandukwala HS An J Lamperti ED Thompson EC Hastie R Ten-eleven-translocation 2 (TET2) negatively regulates homeostasis and differentiation of hematopoietic stem cells in mice. Proc Natl Acad Sci U S A (2011) 108(35):14566–71 10.1073/pnas.1112317108 21873190 PMC3167529

[54] Li Z Cai X Cai CL Wang J Zhang W Petersen BE Deletion of Tet2 in mice leads to dysregulated hematopoietic stem cells and subsequent development of myeloid malignancies. Blood (2011) 118(17):4509–18 10.1182/blood-2010-12-325241 21803851 PMC3952630

[55] Moran-Crusio K Reavie L Shih A Abdel-Wahab O Ndiaye-Lobry D Lobry C Tet2 loss leads to increased hematopoietic stem cell self-renewal and myeloid transformation. Cancer Cell (2011) 20(1):11–24 10.1016/j.ccr.2011.06.001 21723200 PMC3194039

[56] Sigvardsson M Clark DR Fitzsimmons D Doyle M Akerblad P Breslin T Early B-cell factor, E2A, and Pax-5 cooperate to activate the early B cell-specific mb-1 promoter. Mol Cell Biol (2002) 22(24):8539–51 10.1128/MCB.22.24.8539-8551.2002 12446773 PMC139876

[57] Liu Z Widlak P Zou Y Xiao F Oh M Li S A recombination silencer that specifies heterochromatin positioning and ikaros association in the immunoglobulin kappa locus. Immunity (2006) 24(4):405–15 10.1016/j.immuni.2006.02.001 16618599

[58] Popov AV Zou X Xian J Nicholson IC Bruggemann M A human immunoglobulin lambda locus is similarly well expressed in mice and humans. J Exp Med (1999) 189(10):1611–20 10.1084/jem.189.10.1611 10330440 PMC2193639

[59] Gonda H Sugai M Nambu Y Katakai T Agata Y Mori KJ The balance between Pax5 and Id2 activities is the key to AID gene expression. J Exp Med (2003) 198(9):1427–37 10.1084/jem.20030802 14581609 PMC2194241

[60] Tran TH Nakata M Suzuki K Begum NA Shinkura R Fagarasan S B cell-specific and stimulation-responsive enhancers derepress Aicda by overcoming the effects of silencers. Nat Immunol (2010) 11(2):148–54 10.1038/ni.1829 19966806

