# Supplementary material for: A Novel Pax5-Binding Regulatory Element in the Igκ Locus
Source: Front Immunol. 2014 May 23;5:240. doi: 10.3389/fimmu.2014.00240 (PMC4033077; doi:10.3389/fimmu.2014.00240)
Supplement: Supplementary file 1 [file Data_Sheet1.PDF]

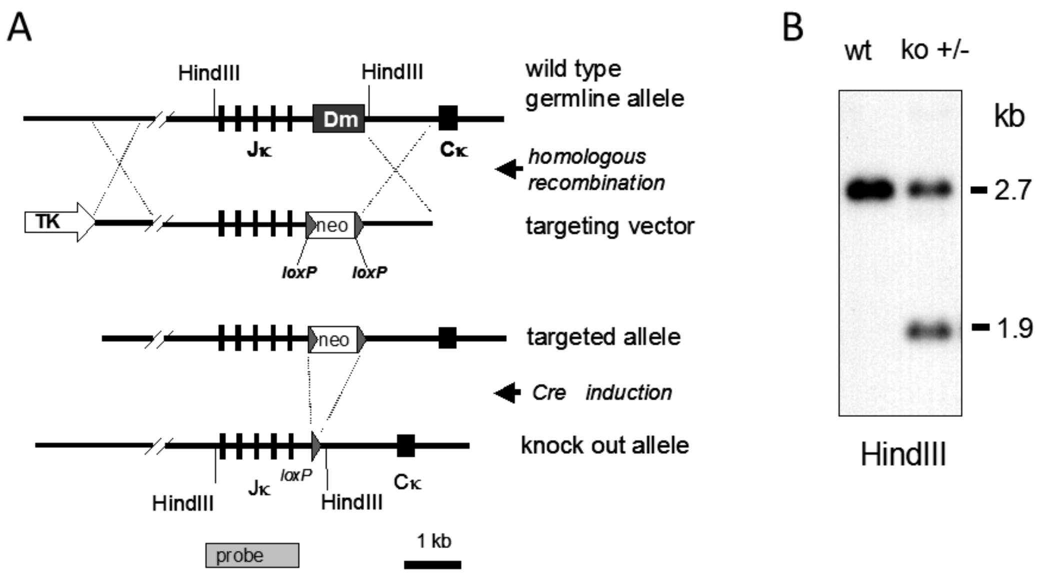


**Supplementary Figure 1 – Targeting strategy for endogenous deletion of Dm element.**

(A) Targeting vector for the Dm element knock out consisted of the germline Igκ gene fragment, with the Dm element replaced with a neomycine resistance cassette, flanked by two loxP recombination sites. Thymidine kinase gene for negative selection was adjacent to the Long Arm of Homology of the construct. The vector was electroporated into ES cells and resistant clones were checked by southern blot for correct allele targeting. Selected targeted clones were transiently transfected with a Cre expressing plasmid. (B) ES clones with the resulted Dm element knock out were confirmed by Southern blot and were used to mice generation. Restriction with HindIII generates a 2.7 kb WT allele fragment and a 1.9 kb ΔDm allele fragment.

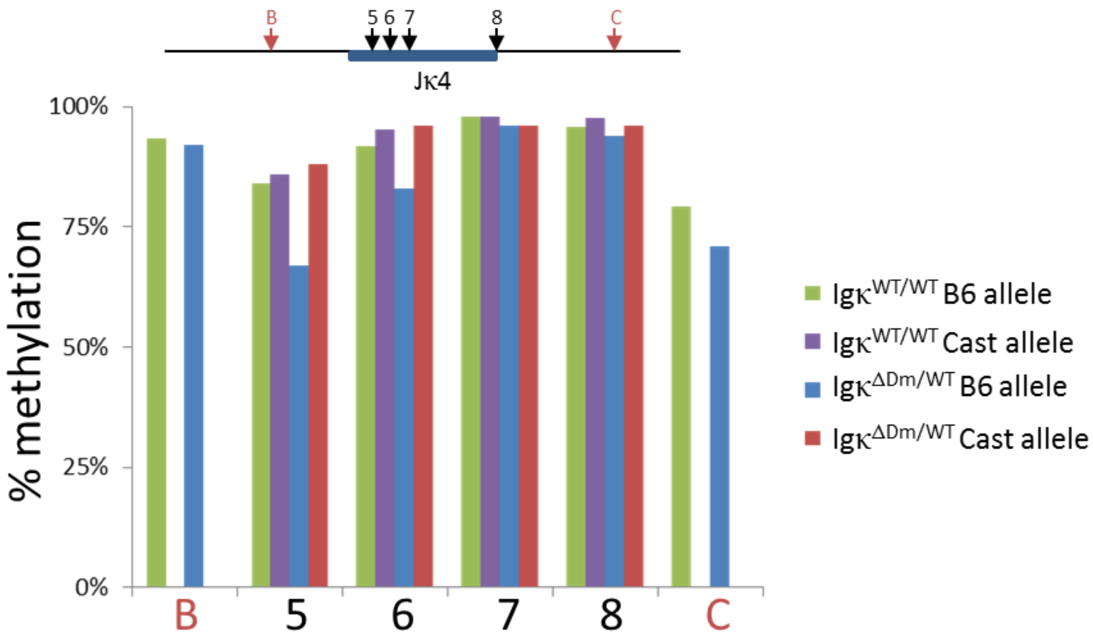


**Supplementary Figure 2 – DNA methylation of the Igκ locus in late pre-B cells of mice containing a deletion of the Dm element.**

Bisulfite treated DNA from $CD19^+$ bone marrow pre-B cells from $Rag1^{-/-}$ C57BL/6/*Casteneous* IgH-3H9-Tg mice with or without a deletion of the Dm element on the C57BL/6 (B6) allele were amplified at the Jκ4 region and sequenced by high-throughput sequencing. 1600-3000 copies of each CpG from each genotype were analyzed. Alleles were differentiated by strain specific polymorphic sites within the amplified regions. The methylation state of each CpG is summarized. Relative locations of CpGs are marked on schematic map. CpGs which are present only in the B6 strain are marked in red.

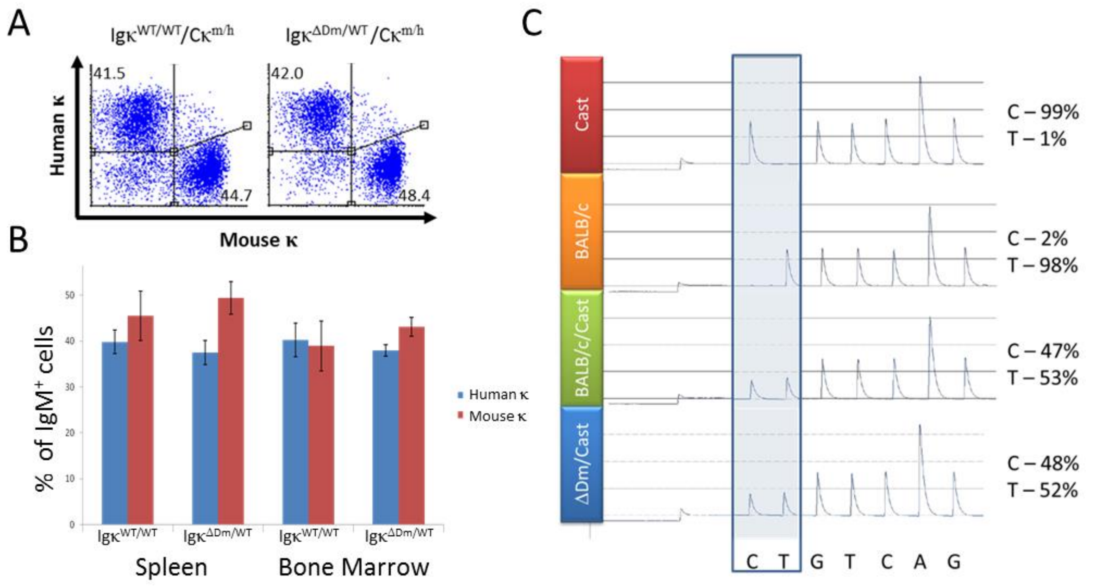


**Supplementary Figure 3 – Rearrangement of the Dm deleted Igκ allele is similar to wildtype allele.**

(A,B) Splenic and bone marrow cells from mice with a knock in of the human Cκ sequence on one allele and either with or without a deletion of the Dm segment on the other allele were stained with αIgM-APC, αhuman-Igκ-FITC and αmouse-Igκ-PE. $IgM^+$ cells were assessed by flow cytometry for human or mouse Igκ expression. (A) Representative FACS plot of human *versus* mouse Igκ of splenic B cells. (B) Summary of human *versus* mouse Igκ positive cells from spleen or bone marrow. Error bars mark standard deviation. 6 mice were analyzed in each group. (C) Rearranged Igκ fragments originating in cDNA from spleens of BALB/c/*Casteneous* mice with or without a deletion of the Dm element on the BALB/c allele were amplified. Allelic distribution of the rearranged molecules was analyzed by pyrosequencing, with alleles being differentiated from each other by a C/T polymorphism in the 3’UTR of the Cκ segment. cDNA from BALB/c and *Castaneous* (Cast) mice were used as controls.

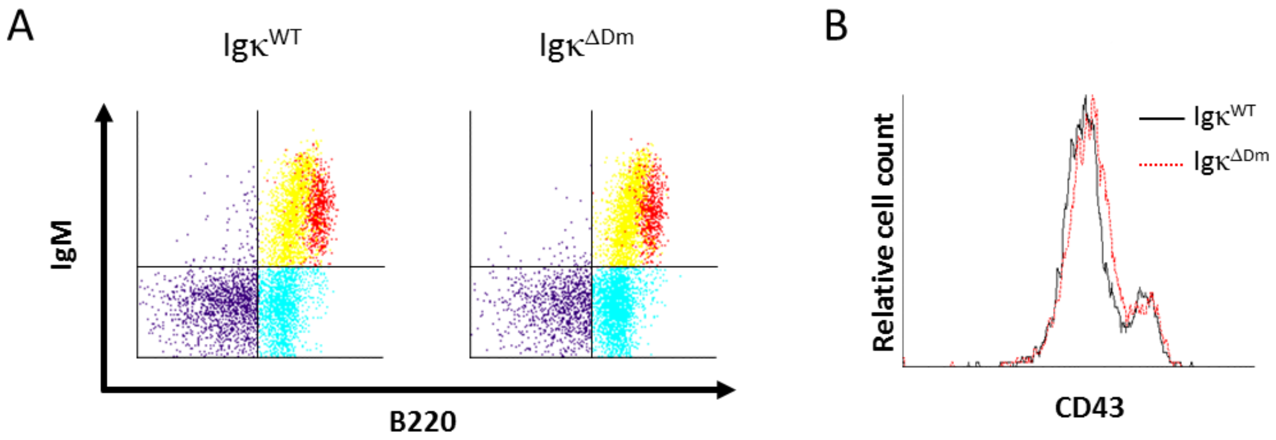


**Supplementary Figure 4 – B cell development in the bone marrow of Dm deficient mice is similar to wild type mice.**

Bone marrow cells from individual Igκ$^{WT/WT}$ or Igκ$^{\Delta Dm/\Delta Dm}$ mice were stained simultaneously with αIgM-APC, αB220-PerCP-Cy5.5, αCD43-PE and αIgD-FITC. B cell populations lying within the lymphocyte gate were quantified by flow cytometry. The B cell populations were defined as: progenitor cells (B220$^+$ IgM$^-$) which subdivided into pro B cells (B220$^+$ IgM$^-$ CD43$^+$) and pre-B cells (B220$^+$ IgM$^-$ CD43$^-$), immature B cells (B220$^+$ IgM$^+$ IgD$^-$) and mature B cells (B220$^+$ IgM$^+$ IgD$^+$). (A) Representative FACS plots from each genotype. Progenitor cells are marked in blue, immature B cells are marked in yellow, mature B cells are marked in red. (B) Representative FACS histogram of CD43 expression in B progenitor cells (Blue gate from (A)).
